# Clinical Significance of Isolated Peri-Appendiceal Lesions in Patients With Left Sided Ulcerative Colitis

**DOI:** 10.4021/gr302w

**Published:** 2011-03-20

**Authors:** Yan Bakman, Jeffry Katz, Chris Shepela

**Affiliations:** a University of Minnesota, Deparment of Internal Medicine, Division of Gastroenterology, MMC 36, 420 Delaware Street SE, Minneapolis, MN 55455, USA; b University Hospitals Case Medical Center, Division of Gastroenterology, 11100 Euclid Avenue Cleveland, Ohio 44106-5066, USA; c Regions Hospital, Digestive Care Center, 435 Phalen Blvd, Saint Paul, MN 55130, USA

**Keywords:** Ulcerative colitis, Peri-appendiceal lesion, Cecal patch, Ulcerative colitis therapy, Inflammatory bowel disease

## Abstract

**Background:**

Ulcerative colitis is classically described as a condition originating in the rectum and extending proximally towards the cecum. In recent years, a discontinuous peri-appendiceal lesion has been described. Our aim was to evaluate the risk of progression to pancolitis in patients presenting with an isolated peri-appendiceal lesion on ileocolonoscopy.

**Methods:**

Endoscopy databases at three tertiary care centers were searched for patients undergoing ileocolonoscopy for diagnosis or surveillance of ulcerative colitis. Patients with isolated periappendiceal lesions as well as histologically confirmed left sided colitis were enrolled. Controls were defined as patients with left-sided ulcerative colitis without evidence of peri-appendiceal inflammation. The main outcome was the need for escalation of therapy to systemic corticosteroids, immunomodulators or biologic agents. Secondary outcomes were progression to pancolitis or requirement for colectomy. A secondary analysis of other risk factors for proximal extension/progression of colitis was also performed.

**Results:**

We identified 228 patients with ulcerative colitis, 123 were included in the analysis. Four point eight percent of patients had isolated peri-appendiceal lesions. In the group with peri-appendiceal lesions, 47.4% required escalation of therapy vs. 70% in the control group (P = 0.53). There was no difference in progression to pan-colitis or colectomy rates between the two groups. Progression was not predicted by inflammatory markers, age, gender, initial Mayo UC score or IBD therapy utilization.

**Conclusions:**

The presence of isolated peri-appendiceal lesions is not a risk factor for future escalation of therapy for ulcerative colitis and is not correlated with proximal extension of disease.

## Introduction

**Figure 1:**
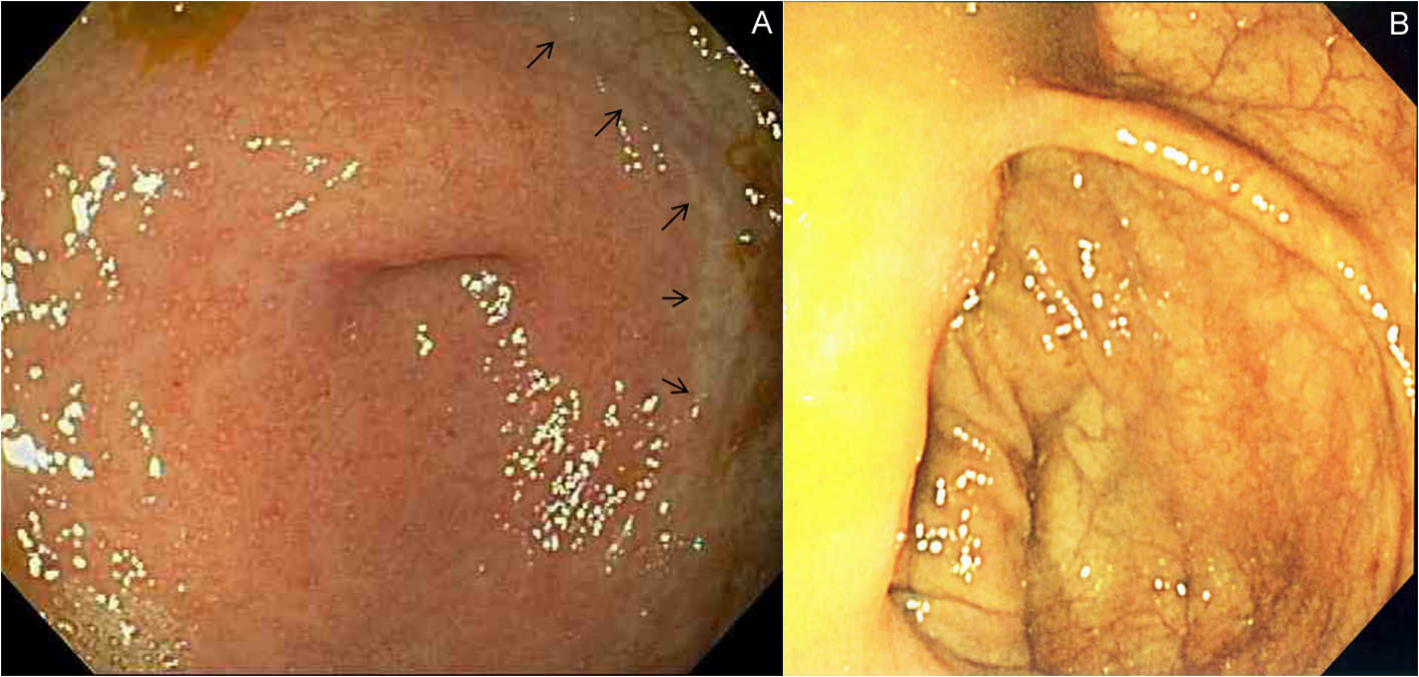
Peri-appendiceal lesion. (A) Typical endoscopic appearance of periappendiceal inflammation, “cecal patch” with a sharply demarcated border (arrows), seen in a patient with left sided ulcerative colitis. (B) Normal endoscopic appearance of the cecum.

Ulcerative colitis (UC) is a chronic inflammatory condition of the colon, the etiology of which is unclear, but is hypothesized to be a complex interplay between the genetic and environmental factors. UC has classically been described as a continuous pattern of disease originating in the rectum and extending without interruption proximally to differing distances in different patients. The condition has a significant impact on the patients’ quality of life typically requiring long term, potentially toxic treatments, often requiring hospitalization, and potentially leading to a variety of complications including toxic megacolon, colectomy, and colorectal cancer. Progression of limited ulcerative colitis to pancolitis has been associated with a more severe disease course requiring more medications, increased risk for colorectal cancer, and an increased risk for colectomy [1, 2]. The ability to assess the risk of progression to ulcerative pancolitis would be a valuable tool in the initial management of the disease, as more aggressive therapies may be required to prevent long term complications in patients known to be at high risk for disease progression. Over the past 30 years, the presence of peri-appendiceal lesions (“cecal patch”) (Fig. 1) in patients with ulcerative pan-colitis has been recognized, although the importance of this finding has been unclear. Multiple series have found an association between surgical removal of the appendix and lower incidence of ulcerative colitis and even resolution of limited colitis [3-5]. To our knowledge no attempt has been made to evaluate the significance of a cecal patch in predicting the severity of UC. In this retrospective cohort study we evaluated the potential association between the presence of a cecal patch on initial ileo-colonoscopy and eventual requirement for colectomy or escalation of medical therapy to systemic steroids, immunomodulators, or biologic agents.

## Methods

We searched a database of all colonoscopies performed at the University of Minnesota Medical Center and the Minneapolis VA Medical Center between 2002 and 2007. All patients undergoing colonoscopy for diagnosis or follow up of ulcerative colitis were considered for the study. We excluded those patients with indeterminate colitis, those with initial extent of the disease proximal to the splenic flexure, lack of follow up colonoscopy, unsuccessful colonoscopy, or inability to clearly visualize the cecum. Additionally, only patients with mild to moderately active disease at the index colonoscopy and those naive to immune modulators and biologic agents were included. Endoscopic reports mentioning a cecal patch, peri-appendiceal inflammation, cecal inflammation in the presence of proctitis or left sided colitis distal to the splenic flexure were considered to have met the inclusion criteria for having a cecal patch. For multivariate analysis, controls were selected in a 2 : 1 ratio and were frequency matched to the cecal patch cohort according to the gender, age of onset (within 10 years), and initial extent of disease. Baseline information including gender, age at diagnosis, erythrocyte sedimentation rate, C-reactive protein, leukocyte count, platelet count, family history, duration of disease, and initial therapy was collected by reviewing the patients’ medical records including outpatient gastroenterology clinic notes, inpatient gastroenterology consults, available records from outside hospitals and clinics, and general medicine clinic notes. The follow up data was collected by reviewing the endoscopic database and the patients’ electronic medical record.

For multivariate analysis, additional patients with limited ulcerative colitis and a cecal patch were contributed by the University Hospitals Case Medical Center (UHCMC). These additional patients were identified through search of an IBD database maintained at UHCMC. The database of 969 IBD patients was queried for a diagnosis of “proctitis”, “proctosigmoiditis” and “cecal patch”. Controls were selected from the same database matched for gender, age of onset (within 10 years), and initial extent of disease. The institutional review board at all centers approved the study protocol and design.

The primary outcome was the need for escalation of maintenance therapy to include either recurrent pulses of systemic corticosteroids, or initiation of immunomodulators or biologic agents. Secondary outcomes included progression to pan-colitis and uncontrolled disease requiring colectomy. A multivariate regression analysis was performed on the data collected on patients at the University of Minnesota and the Minneapolis VA Medical Center to assess whether the patients’ gender, age at diagnosis, Mayo endoscopic severity score at time of diagnosis, family history of inflammatory bowel disease, initial laboratory values, and initial therapy were associated with escalation of medical therapy or requirement for surgery.

## Results

We reviewed the reports for 228 patients undergoing endoscopic procedures having a diagnosis of “colitis”. One hundred fifty six of these were cared for at the University of Minnesota Medical Center, and 72 at the Veterans Medical Center. One hundred and five patients were excluded: 33 patients had no follow up data vailable, 59 patients had pan-colitis on presentation, 2 patients had a prior colectomy, and 11 patients lacked initial colonoscopy data. Among the included 123 patients we identified 11 patients with a pathologically proven cecal patch, resulting in an overall prevalence of 4.8 percent. The cases had a mean follow-up of 68.7 months. An additional 9 patients were identified at the University Hospitals Case Medical Center. From a database of 969 IBD patients, 231 with confirmed ulcerative colitis were identified. Of these, 9 patients were found to have a diagnosis of ulcerative colitis with a pathologically proven cecal patch, for a prevalence rate of 3.8 percent. The cases had a mean follow-up of 55 months.

**Figure 2:**
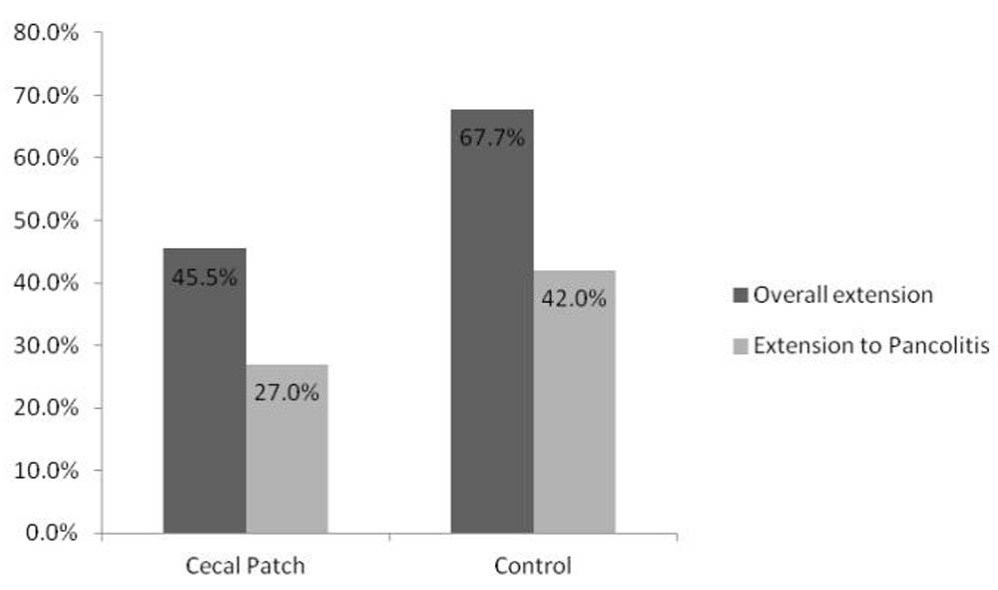
Rates of endoscopic disease progression.

**Figure 3:**
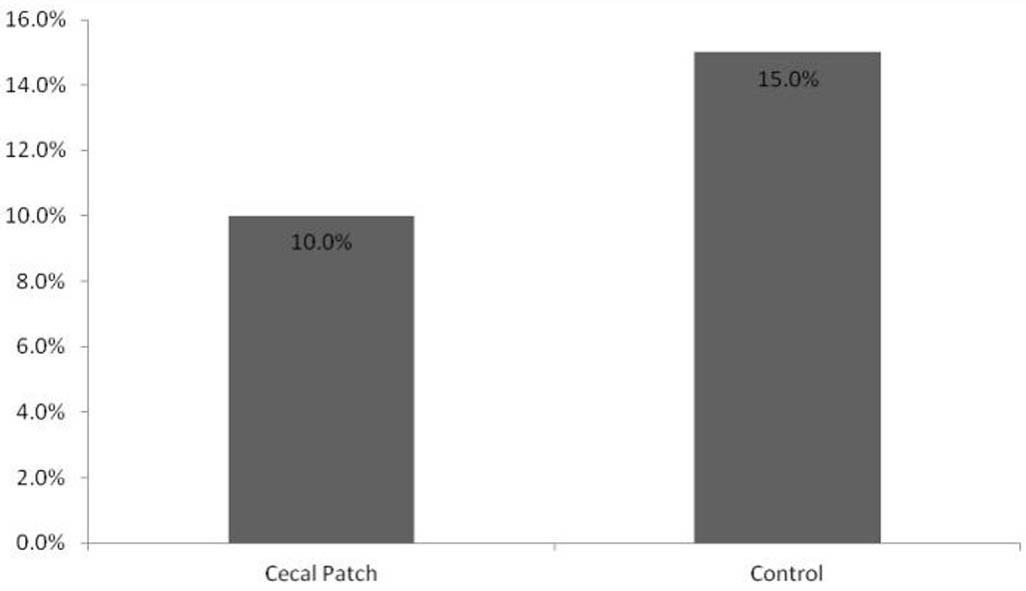
Rates of colectomy between groups.

The cases and controls were well matched in regards to family history, Mayo endoscopic severity score, platelet count, leukocyte count, follow up time, and the initial therapy required (Table 1). Nine patients did not have sufficient follow up data to assess disease progression. Of those patients in whom follow up data was available, in the group with a cecal patch, 47.4% of the patients required escalation of therapy vs. 70% of the controls (P = 0.53). The rate of progression to pan-colitis in the cecal patch group was 27% vs. 42% (P = 0.49) in the control group (Fig. 2). The requirement for colectomy was 10% vs. 15%, P = 0.75 respectively in the cecal patch and control groups (Fig. 3).

**Table 1:** Baseline Characteristics of Patients With and Without a Cecal Patch

	Patients With Extension (N = 33)	Patients Without Extension (N = 90)	Unadjusted Odds Ratio (95% CI)
	number (percent)	number (percent)	
Sex			
Female	20 (60.6)	69 (76.7)	2.1 (0.9 - 5.0)
Male	13 (39.4)	21 (23.3)	
Mean age at diagnosis	32.9	38.4	1.0 (1.0 - 1.0)
Mean ESR	32.2	20.5	1.0 (1.0 - 1.0)
Mean CRP	16.7	17.2	1.0 (1.0 - 1.0)
Initial Mayo endoscopic severity score			
1	12 (36.4)	48 (55.8)	2.2 (1.0 - 5.1)
2 or more	21 (63.6)	38 (44.2)	
Mean WBC	8,033.3	7,864.1	1.0 (1.0 - 1.0)
Mean platelets	319	298.4	1.0 (1.0 - 1.0)
Family history of UC	3 (9.1)	2 (2.2)	3.5 (0.6 - 22.5)
Initial antibiotic use	3 (9.4)	3 (3.6)	2.8 (0.5 - 14.6)
Initial mesalamine suppository use	10 (31.3)	17 (20.0)	1.8 (0.7 - 4.6)
Initial steroid suppository use	4 (12.5)	11 (12.9)	1.0 (0.3 - 3.3)
Initial mesalamine/sulfa use	25 (78.1)	70 (82.4)	0.8 (0.3 - 2.1)
Initial oral steroid use	16 (50.0)	32 (37.7)	1.7 (0.7 - 3.8)

A proximal extension of UC was noted in 26.8% (33/123) of patients in the cecal patch group over the time of study. In a multivariate analysis of the characteristics of those patients extending their disease to pan-colitis, the initial Mayo endoscopic severity index and male gender approached statistical significance as a risk factor for proximal extension of UC however no other statistically significant risk factors were found (Table 2).

**Table 2:** Covariates for Proximal Extension of Ulcerative Colitis

	Cecal Patch (N = 20)	Control (N = 40)	P-value
	number (percent)	number (percent)	
Family History	4 (20)	7 (17.5)	NS
Laboratory Data			
Platelets	316.9	323.8	NS
WBC	4,535	4,440	NS
ESR	23.2	23.4	NS
C-reactive protein	15.5	12.0	NS
Initial Therapy			
Antibiotics	3 (15)	12 (20)	NS
5-ASA suppository	11 (55)	22 (55)	NS
Steroid suppository	5 (25)	18 (45)	NS
Oral ASA product	15 (75)	35 (87.5)	NS
Oral steroids	5 (27.8)	22 (55.0)	0.056
Mayo Endoscopic Severity Score			
0	2 (10.5)	6 (15.0)	NS
1	11 (57.9)	17 (42.5)	NS
2 - 4	6 (31.6)	17 (42.5)	NS
Initial Extent			
Proctitis	11 (55)	11 (27.8)	0.049
Left-sided colitis	9 (45)	29 (72.5)	0.049
Age at Diagnosis	27.6	30.7	NS
Mean Follow Up Time (months)	63.3	71.4	NS

## Discussion

We performed a multi-center retrospective cohort study to evaluate the prevalence of the cecal patch in patients with left sided ulcerative colitis or ulcerative proctitis as well as its clinical significance and value in predicting the need for escalation of therapy. Our data show that the cecal patches are rare with an overall prevalence of less than 5% in a patient population seen at the Minneapolis VA, University of Minnesota Medical Center, and University Hospitals Case Medical Center. Furthermore, the presence of a cecal patch does not predict the need for escalation of therapy, greater extension to pan-colitis or higher colectomy rates. A multivariate analysis did not reveal any other factors that were associated with escalation of therapy or progression of disease.

The initial report by Cohen in 1974 described a case of a skipped peri-appendiceal lesion in a patient with distal ulcerative colitis [6]. Since then, multiple reports have appeared confirming the presence of such “skipped lesions” in the setting of distal ulcerative colitis, and assessing their clinical significance. The incidence of such lesions has been estimated to be between 21% and 86% by colectomy studies and as much as 24% - 58% by endoscopic studies [7-10]. Very few trials describing the clinical significance of peri-appendiceal lesions exist. A trial by Matsumoto et al. suggested that the presence of a peri-appendiceal lesion indicates more severe distal disease and better response to therapy [8]. Most recently, a prospective trial by Byeon et al. showed that the presence of such lesions did not predict relapse, proximal extension, or need for procto-colectomy [11].

Numerous studies, however, have established a role for the appendix in the immunopathophysiology of ulcerative colitis, showing that patients who have previously undergone appendectomy are at much lower risk of developing ulcerative colitis as compared to the general population. Most of these studies have demonstrated odds ratios of 0.2 - 0.3 [12-15].

We observed a much lower incidence of peri-appendiceal lesions than what has previously been reported. This lower incidence was consistent in data from both the University of Minnesota/Minnapolis VA as well as the University Hospital Case Medical Center. One potential reason for this was our use of a very strict definition of a peri-appendiceal lesion in that both endoscopic and histologic features had to be present. Most other trials have used either exclusively pathologic or endoscopic features to establish the diagnosis. This may have increased the prevalence observed in those studies. In fact, one study observed an absence of endoscopic findings in up to 24% of patients with pathologically proven peri-appendiceal lesions [7]. Furthermore, the prevalence of peri-appendiceal lesions may be affected by previous therapy as pointed out by Bernstein et al. [16]. Patients undergoing colectomy have usually failed medical therapy and thus may have peri-appendiceal lesions on colectomy specimens. Our findings that peri-appendiceal lesions do not correlate with more severe disease or extension to pan-colitis are in concordance with the study by Byeon et al., the only previous trial evaluating this outcome. In contrast to that study, however, we have used a stricter definition of periappendiceal lesion, included patients with lesions diagnosed at any time during the follow up, and had a larger sample size. We could not replicate the findings of Matsumoto et al., that periappendiceal lesions predict better responsiveness to therapy.

Our study has a number of limitations. The observed incidence of a cecal patch was low, therefore, despite searching the databases at three institutions. A 30% increased incidence of progression to pan-colitis would be required in the cecal patch group for our study to reach statistical significance. Therefore, a smaller effect could have been missed. Furthermore, the observed incidence of extension to pan-colitis was substantially higher than expected in the control group, possibly reflecting the complex patient population seen at our tertiary referral centers. Since our trial was a retrospective analysis, we cannot prove cause and effect. Multiple endoscopists performed the procedures and were not specifically looking for a peri-appendiceal lesion. This introduces significant inter-observer variability, and subtle findings may have been missed. This is in contrast to the previous studies where such lesions were specifically sought after, and only a limited number of endoscopists were involved. The multicenter design allows for a larger number of patients, but introduces some heterogeneity in the data. As an example the VA population is predominantly male, and has a high incidence of other comorbidities. We have attempted to minimize the impact of this factor by matching cases to controls from the same institution. The follow up time for patients with a cecal patch tended to be longer than that for study subjects without this finding, although this was not statistically significant. It is possible that with longer follow up a higher incidence of pancolitis could be discovered in the control group. However, this would not impact the finding that a cecal patch is not a risk factor for endoscopic progression of disease. Finally, given the retrospective nature of the study, there was no uniform approach to initial patient care, and some baseline laboratory values were not available for all patients. It is possible that initial management influenced the prevalence of the peri-appendiceal lesions, a theory that warrants further investigations in future, controlled trials.

In conclusion, in our analysis, the incidence of peri-appendiceal inflammation in patients with ulcerative proctitis, proctosigmoiditis, or left-sided UC is lower than previously reported. We have found no evidence that finding a cecal patch in patients with UC impacts the clinical course or influences disease management. We did not identify any predictors of disease progression or severity associated with a peri-appendiceal cecal patch. Further prospective studies are needed to clarify the incidence of these lesions and to further assess their relationship to initial management of the UC.
